# Supplementary material for: The genome sequence of the Notch-wing Button, Acleris emargana (Fabricius, 1775)
Source: Wellcome Open Res. 2023 Aug 30;8:376. [Version 1] doi: 10.12688/wellcomeopenres.19843.1 (PMC11170068; doi:10.12688/wellcomeopenres.19843.1)
Supplement: Peer review history

# Reviewer report: Reviewer response for version 1

**DOI:** 10.21956/wellcomeopenres.21975.r84318

**Published:** 2024-06-12

**Authors:** Bingzhong Ren

**Affiliations:** 1 Northeast Normal University, Changchun, China

The paper presents an outstanding genomic dataset of *Acleris emargana*, with detailed elaboration on the species' background and a comprehensive summary of the generated genome. Overall, this is an excellent genomic dataset, but there are a few minor comments:

 Q1. The authors mentioned in the research background that *Acleris emargana* is often confused with *Acleris effractana*. In this case, it is suggested to provide criteria for identification. Evidence should be given from either morphological or genetic levels to confirm that the sequenced species is indeed *Acleris emargan*a.

 Q2. The sequencing methods section should detail the sequencing strategy.

Are sufficient details of methods and materials provided to allow replication by others?

Yes

Is the rationale for creating the dataset(s) clearly described?

Yes

Are the datasets clearly presented in a useable and accessible format?

Yes

Are the protocols appropriate and is the work technically sound?

Yes

Reviewer Expertise:

Insect resources and their utilization; Insect systematics

I confirm that I have read this submission and believe that I have an appropriate level of expertise to confirm that it is of an acceptable scientific standard.

# Reviewer report: Reviewer response for version 1

**DOI:** 10.21956/wellcomeopenres.21975.r84317

**Published:** 2024-06-05

**Authors:** Konrad Fiedler

**Affiliations:** 1 University of Vienna, Vienna, Austria

This paper presents a thorough solid technical report on the genome assembly of a tortricid moth from the UK, identified as Acleris emargana. My only question to this report is the way how the sequenced specimen has been identified in terms of taxonomy. This must be explicitly documented in the paper, since it is now well known that the taxon in question belongs to a species complex formed by 2 (if not even 3?) species.

 The only reliable way to distinguish *emargana* from its sister *taxon effractana* is by anatomy of the genitalia, or by DNA barcoding. Please say clearly which way you have used to identify our focal specimen – since from the time of publication onwards the genomic information will be linked to the respective taxon name ‘forever’. Hence, identification should be 100% error-free.

 In this respect, it is noteworthy that the complex around *Acleris emargana/effractana* comprises 3 clusters in the bold systems database:

 BOLD:AAB3524 (Europe; *Acleris emargana*); BOLD:AAB3525 (North America, Europe; A. effractana); BOLD:AAA6999 (Europe),

 with specimens of *effractana* also being (marginally) further differentiated between North American and European origin (see here, Giligan et al. 2020 (Ref -1): https://doi.org/10.3390/insects11090594).

 Because of these circumstances, it may also be noted in the paper that all information (e.g. on host plants) pre-dating the recognition of *effractana* as a distinct species (pre-2005) is a bit suspect, as long as no voucher specimens exist, whose taxonomic identity has been clarified in retrospect. Therefore, any paragraphs where ecological on *Acleris emargana* are mentioned in this paper reporting its genome should be restricted to sources in which the taxonomic identity has been rigorously checked.

 Otherwise: a valid technical report that makes available important genomic data to the scientific community.

Are sufficient details of methods and materials provided to allow replication by others?

No

Is the rationale for creating the dataset(s) clearly described?

Yes

Are the datasets clearly presented in a useable and accessible format?

Yes

Are the protocols appropriate and is the work technically sound?

Yes

Reviewer Expertise:

Ecology, conservation and biodiversity of insects, with special emphasis on the Lepidoptera

I confirm that I have read this submission and believe that I have an appropriate level of expertise to confirm that it is of an acceptable scientific standard, however I have significant reservations, as outlined above.

## References

[1] : Immigrant Tortricidae: Holarctic versus Introduced Species in North America. *Insects* .2020;11(9) : 10.3390/insects11090594 10.3390/insects11090594 32899282 PMC7564570

# Reviewer report: Reviewer response for version 1

**DOI:** 10.21956/wellcomeopenres.21975.r78304

**Published:** 2024-05-07

**Authors:** Sabtharishi Subramanian

**Affiliations:** 1 ICAR- Indian Agricultural Research Institute, Pusa, New Delhi, India

This paper describes the genome assembly of a genome assembly of Notch-wing Button moth , *Acleris emargana *;(Insecta; Arthropoda Lepidoptera; Tortricidae). A. *emargana i*s widely found cross northern Eurasia east to at least northern China and Altai and Buryatia in Russia. The genome sequence is 691.4 megabases in span scaffolded into 30 chromosomal pseudomolecules, including the Z sex chromosome. The mitochondrial genome has also been assembled and is 16.34 kilobases in length. Gene annotation of this assembly on Ensembl identified 21,886 protein coding genes.

 Queries:

 Q1. Since this moth's taxonomic identity has resemblance to *Acleris effractana, *how the authors can authentically say that the moth collected was actually *Acleris emargana?*

 Whether voucher specimen has been deposited with any repository.

 Q2. Whether barcoding was done for the specimen collected and accession number was obtained from Barcode of Life data base or NCBI GenBank?

Are sufficient details of methods and materials provided to allow replication by others?

Yes

Is the rationale for creating the dataset(s) clearly described?

Yes

Are the datasets clearly presented in a useable and accessible format?

Yes

Are the protocols appropriate and is the work technically sound?

Yes

Reviewer Expertise:

Entomology - Insect Molecular Biology

I confirm that I have read this submission and believe that I have an appropriate level of expertise to confirm that it is of an acceptable scientific standard.
